# Effects of sodium-glucose transporter-2 inhibition on systemic hemodynamics, renal function, and intra-renal oxygenation in sepsis-associated acute kidney injury

**DOI:** 10.1186/s40635-024-00647-2

**Published:** 2024-07-08

**Authors:** Abraham H. Hulst, Connie P. C. Ow, Clive N. May, Sally H. Hood, Mark P. Plummer, Jeroen Hermanides, Daniël H. van Raalte, Adam M. Deane, Rinaldo Bellomo, Yugeesh R. Lankadeva

**Affiliations:** 1 Preclinical Critical Care Unit, Florey Institute of Neuroscience and Mental Health, The University of Melbourne, Melbourne, VIC Australia; 2 Department of Anesthesiology, Amsterdam UMC, University of Amsterdam, Meibergdreef 9, 1105 AZ Amsterdam, The Netherlands; 3 Department of Intensive Care, Royal Adelaide Hospital, Adelaide, Australia; 4 Department of Internal Medicine, Amsterdam UMC, University of Amsterdam, Amsterdam, The Netherlands; 5 Department of Intensive Care, Royal Melbourne Hospital, Melbourne, VIC Australia; 6 Department of Critical Care, Melbourne Medical School, The University of Melbourne, Melbourne, VIC Australia; 7 Australian and New Zealand Intensive Care Research Centre, Monash University, Melbourne, VIC Australia; 8 Department of Intensive Care, Austin Hospital, Melbourne, VIC Australia; 9 Department of Anesthesia, Austin Hospital, Melbourne, VIC Australia

**Keywords:** Sodium-glucose transporter 2 inhibitor, Empagliflozin, Sepsis, Acute kidney injury, Medullary oxygenation

## Abstract

**Background:**

People with type 2 diabetes mellitus treated with sodium-glucose transporter-2 inhibitors (SGLT2i) have lower rates of acute kidney injury (AKI). Sepsis is responsible for the majority of AKI in critically ill patients. This study investigated whether SGLT2i is renoprotective in an ovine model of Gram-negative septic AKI.

**Methods:**

Sixteen healthy merino ewes were surgically instrumented to enable measurement of mean arterial pressure, cardiac output, renal blood flow, renal cortical and medullary perfusion, and oxygenation. After a 5-day recovery period, sepsis was induced via slow and continuous intravenous infusion of live *Escherichia coli*. Twenty-three hours later, sheep were randomized to receive an intravenous bolus of 0.2 mg/kg empagliflozin (n = 8) or a fluid-matched vehicle (n = 8).

**Results:**

Empagliflozin treatment did not significantly reduce renal medullary hypoperfusion or hypoxia, improve kidney function, or induce histological changes. Renal cortical oxygenation during the intervention period was 47.6 ± 5.9 mmHg in the empagliflozin group compared with 40.6 ± 8.2 mmHg in the placebo group (*P* = 0.16). Renal medullary oxygenation was 28.0 ± 18.5 mmHg in the empagliflozin compared with 25.7 ± 16.3 mmHg (*P* = 0.82). Empagliflozin treatment did not result in significant between-group differences in renal blood flow, kidney function, or renal histopathological changes.

**Conclusion:**

In a large mammalian model of septic AKI, a single dose of empagliflozin did not improve renal microcirculatory perfusion, oxygenation, kidney function, or histopathology.

**Supplementary Information:**

The online version contains supplementary material available at 10.1186/s40635-024-00647-2.

## Introduction

Sepsis is the leading cause of acute kidney injury (AKI) in intensive care units (ICU) [1]. Sepsis-associated AKI (SA-AKI) worsens prognosis compared with either disease alone [1, 2]. SA-AKI is associated with a prolonged length of stay in ICU, higher mortality, greater propensity to develop chronic kidney disease, and reduced quality of life [3–6]. The consensus amongst intensivists on managing patients at risk of AKI due to sepsis are the following: preservation of tissue oxygenation, correction of hypovolemia and hypotension, and avoiding nephrotoxins. However, apart from these generalized interventions, no specific targeted protective therapies exist to treat or prevent AKI in patients with sepsis [7].

Sodium-glucose transporter-2 inhibitors (SGLT2i) are a relatively novel class of medication, initially developed to reduce hyperglycemia in patients with type 2 diabetes mellitus (T2D). However, SGLT2i has been shown to have profound cardiovascular and kidney-protective effects in people with and without type 2 diabetes mellitus, including a reduction in heart failure hospitalizations and progression of chronic kidney disease [8–11]. Additionally, post hoc analysis of these large cardiovascular outcome trials also detected SGLT2i-associated reductions in AKI incidences [12–15].

Several mechanisms conferring protection from AKI have been proposed, including a diuretic effect through inhibition of the tubuloglomerular feedback mechanism and its anti-inflammatory effects [16]. Glucose reabsorption in the kidney is coupled with the active reabsorption of sodium in the proximal tubule. It has been proposed that inhibition of SGLT2 will reduce renal oxygen consumption and thereby contribute to the alleviation of renal tissue hypoxia, a key pathophysiological feature of AKI, and the development of chronic kidney disease [17–19]. Given the pressing need for an effective intervention for SA-AKI and the promising trial data in reducing AKI with longer-term SGLT2i treatment, we aimed to investigate whether a single dose of SGLT2i, at a clinically relevant dosage, can alleviate renal tissue hypoxia in an ovine model of Gram-negative SA-AKI. We hypothesized that empagliflozin, an SGLT2i, would improve renal cortical and medullary tissue oxygenation and kidney function in sheep with established septic AKI.

## Materials and methods

### Animals

Sixteen female merino ewes (35–45 kg body weight) were housed in individual metabolic cages with free access to water and 800 g/day oaten chaff. The Animal Ethics Committee of the Florey Institute of Neuroscience and Mental Health (Ethics identification number: 21-030-FINMH) approved these experiments under guidelines laid down by the National Health and Medical Research Council of Australia. This report was written in accordance with the ARRIVE 2.0 guidelines [20]. As previously described, the ewes underwent two aseptic surgical procedures, including the general anesthetic techniques, as summarized below [21–30]. During the first surgery, the left carotid artery was exteriorized into a skin fold to form a carotid arterial loop, allowing easy access for subsequent arterial cannulation [24, 28]. Subsequently, a 20-mm transit-time flow probe (Transonic Systems, Ithaca, NY) was placed around the pulmonary artery to measure cardiac output (CO) [24]. Three weeks later, the carotid artery was cannulated, and the catheter was connected to a pressure transducer for measurement of arterial blood pressure (ABP) (providing systolic, diastolic, and mean arterial pressure: SBP, DBP, MAP) and heart rate and for collection of blood samples [28]. Three catheters were inserted into the right jugular vein: one for delivery of treatment, one for administering *E. coli*, and one for fluid resuscitation and vasopressors, as required. To maintain patency, the arterial and venous catheters were continuously infused with heparinized saline (10 U heparin/mL at 3 mL/hr). The next day, in the second surgical procedure, a 4-mm transit-time flow probe (Transonic Systems) was placed around the left renal artery to measure renal blood flow (RBF) [24]. The renal vein was cannulated for blood sampling, and two fiber-optic probes (Oxford Optronix, Abingdon, United Kingdom) were inserted into the renal cortex and medulla to measure renal cortical and medullary perfusion (RCP, RMP) and oxygenation (PrcO_2_, RrmO_2_) [22, 29]. A Foley catheter was inserted into the bladder, and a fiber-optic probe was inserted through a port and advanced to the tip of the Foley catheter to continuously measure partial urinary oxygen pressure (PuO_2_) [21, 22]. For all surgical procedures, animals were injected with intramuscular antibiotics (900 mg procaine penicillin, Ilium Propen, Troy Laboratories, Smithfield, NSW, Australia) and an analgesic (Flunixin meglumine, 1 mg/kg; Troy Laboratories or Mavlab), at start of surgery prior to the first incision and at 24 and 48 h postsurgery [22, 25, 30]. Before the experiments, animals were allowed at least five days of recovery following the second surgical procedure to minimize any effects of surgical stress.

### Experimental protocol

**Fig. 1:**
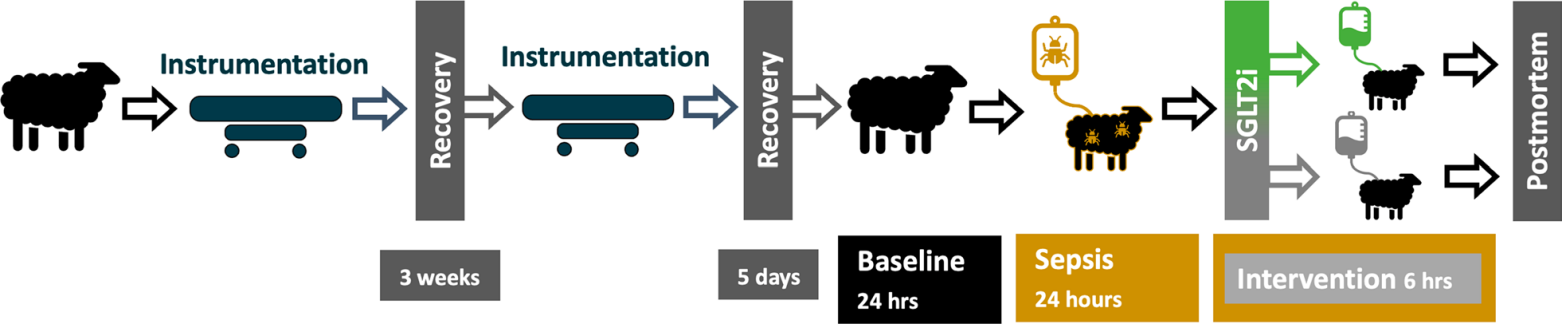
Study overview and experimental timeline

A schematic representation of the experimental protocol and data collection time points are depicted in Fig. 1. Following a 24-h baseline period, sepsis was induced in non-anesthetized sheep with an intravenous dose of live *E. coli* (2.8 × 10^9^ colony-forming units [CFUs] over 30 min) as a bolus, followed by a continuous infusion (1.26 × 10^9^ CFU/h for the rest of the experiment). At 23.5 h of sepsis, fluid bolus therapy with Hartmann solution (Baxter Australia, 30 mL/kg over 30 min) was administered [30]. At 24 h of sepsis, the animals were randomized by online software built into the electronic data capturing software (Castor EDC, Castor B.V., Amsterdam, Netherlands). Animals in the intervention group received an IV bolus of 0.2 mg/kg empagliflozin (MedChemExpress LLC, Monmouth Junction, NJ, USA) dissolved in ß-cyclodextrin (MedChemExpress LLC, Monmouth Junction, NJ, USA) over 10 min. This is a clinically effective dose, as indicated by previous studies in critically ill patients in which no major adverse events were reported [31]. Animals in the comparator group received an equal volume of vehicle solution with ß-cyclodextrin. At the end of the protocol, 6 h after the bolus treatment, animals were euthanized with a lethal dose of sodium pentobarbitone (100 mg/kg, IV). Positions of the renal fiber-optic probes were confirmed at autopsy, and kidney biopsies were taken for histopathological assessment. [30]

### Data collection

A computer with a CED 1401 interface, running a data acquisition system (Spike 2 Software, Cambridge Electronic Design, Cambridge, United Kingdom), continuously recorded analog signals (ABP, heart rate, CO, renal blood flow, RCP, RMP, PrcO_2_ and RrmO_2_), temperature, and PuO_2_ at 100 Hz. Renal vascular conductance (RVC) was calculated as RBF/MAP. Stroke volume (SV) was calculated as CO/heart rate. We calculated body surface area (BSA) as 0.09*Weight^(0.67)^, and cardiac index (CI) and stroke volume index (SVI) were calculated as CO/BSA and SV/BSA, respectively. We recorded hourly urine flow and collected 1-hourly urine samples at baseline and at 24, 26, 28, and 30 h time-points after induction of sepsis. Urine samples were collected for measurement of creatinine and sodium concentrations and subsequent analysis of renal excretory function. Arterial and renal venous blood samples were collected at baseline, just prior to the infusion of E. coli, and subsequently at 24, 26, 28, and 30 h of sepsis for measurement of blood oximetry (ABL System 625, Radiometer Medical, Copenhagen, Denmark), as well as creatinine, glucose, and ketones. The occurrence of AKI was based on “the Kidney Disease: Improving Global Outcomes (KDIGO)” clinical criteria; stage 1 AKI is characterized by a > 1.5-fold increase in plasma creatinine or oliguria of 0.5 ml/kg/h for > 6 h.

### Statistical analysis

Data are reported as mean ± SD and between-group differences are reported as the difference with a 95% confidence interval (95% CI). MAP, heart rate, RBF, RVC, and biochemical markers are reported as the average over the baseline period and as hourly averages from 24 to 30 h after commencing the infusion of *E. coli*. Given the critical role in the development of AKI, we defined renal medullary tissue oxygenation as the primary outcome [26]. Based on our previous investigations, detecting a 50% reduction in medullary tissue oxygenation with 90% power and α = 0.05 required a sample size of eight sheep per group [26]. Data were analyzed using repeated-measures analysis of variance (ANOVA) with factors Intervention (P_Intervention_: vehicle or empagliflozin), time (P_Time_), and their interaction (P_Intervention*Time_). Specific post-hoc comparisons were made using the Student’s T-test. The absolute changes in response to the intervention after induction of sepsis were compared with responses at baseline, prior to induction of sepsis, and 6 h after treatment commenced, using repeated-measures ANOVA. The histological assessment of kidney tissues collected was scored by a pathologist who was blinded to the intervention and analyzed using Fisher’s exact test. Statistical analysis was performed using GraphPad PRISM 6.0 (GraphPad Software, La Jolla, CA). All variables were assessed for normality and log-transformed where appropriate. A two-sided *P*-value less than or equal to 0.05 was considered statistically significant without correction for multiple comparisons.

## Results

The body weight of sheep treated with empagliflozin (38.9 ± 1.6 kg; n = 8) was similar to that of those treated with vehicle (39.4 ± 1.2 kg; n = 8). There were no significant time effects on the cardiovascular and renal variables during the 24-h baseline period (P time > 0.05), and the basal levels were similar in the two groups (Table 1). No animals died during the period 10–30 h after the infusion of *E. coli* commenced. Therefore, no animals were excluded from the analysis.

**Table 1:** Changes in systemic hemodynamics, global and regional kidney perfusion, oxygenation, and renal function from baseline (premorbid) to 24 h of gram-negative sepsis in non-anesthetized sheep in both treatment groups

Systemic and renal variables	Sheep prior to intervention (n = 16)	
	Baseline	24 h sepsis
Mean arterial pressure (MAP, mmHg)	89 ± 9	77 ± 11*
Heart rate (bpm)	75 ± 13	137 ± 28*
Cardiac output (CO, l/min)	4.1 ± 0.8	6.1 ± 1.5*
Systemic vascular resistance (SVR, mmHg/ml/min)	23 ± 5.6	14 ± 7.5*
Urine Output (UO, ml/kg/h)	1.39 ± 0.57	0.49 ± 0.29*
AKI grade 1 (UO < 0.5ml/kg/h for > 6 h)	0/16	14/16*
Creatinine clearance (ml/min)	65 ± 31	48 ± 29*
Plasma creatinine (µmol/l)	65 ± 7	110 ± 29*
Plasma lactate (mmol/l)	0.50 ± 0.13	1.36 ± 0.84*
Arterial oxygen tension (PaO_2,_ mmHg)	96 ± 8	85 ± 14*
Fractional sodium excretion (FENa, %)	1.2 ± 0.6	0.5 ± 0.4*
Renal blood flow (RBF, ml/min)	276 ± 95	358 ± 135*
Renal oxygen delivery (RDO_2_, ml O_2_/min)	36 ± 11	42 ± 19
Renal oxygen consumption (RVO_2_, ml O_2_/min)	4.8 ± 1.9	3.9 ± 2.8
Renal cortical tissue perfusion (RCP, BPU)	1707 ± 1038	2253 ± 1383
Renal medullary tissue perfusion (RMP, BPU)	750 ± 478	525 ± 524
Renal cortical oxygen tension (PrcO_2_, mmHg)	38 ± 11	45 ± 10
Renal medullary oxygen tension (PrmO_2_, mmHg)	32 ± 17	22 ± 20*

*P < 0.05 comparison 24h sepsis to baseline

### Systemic hemodynamic function

**Fig. 2:**
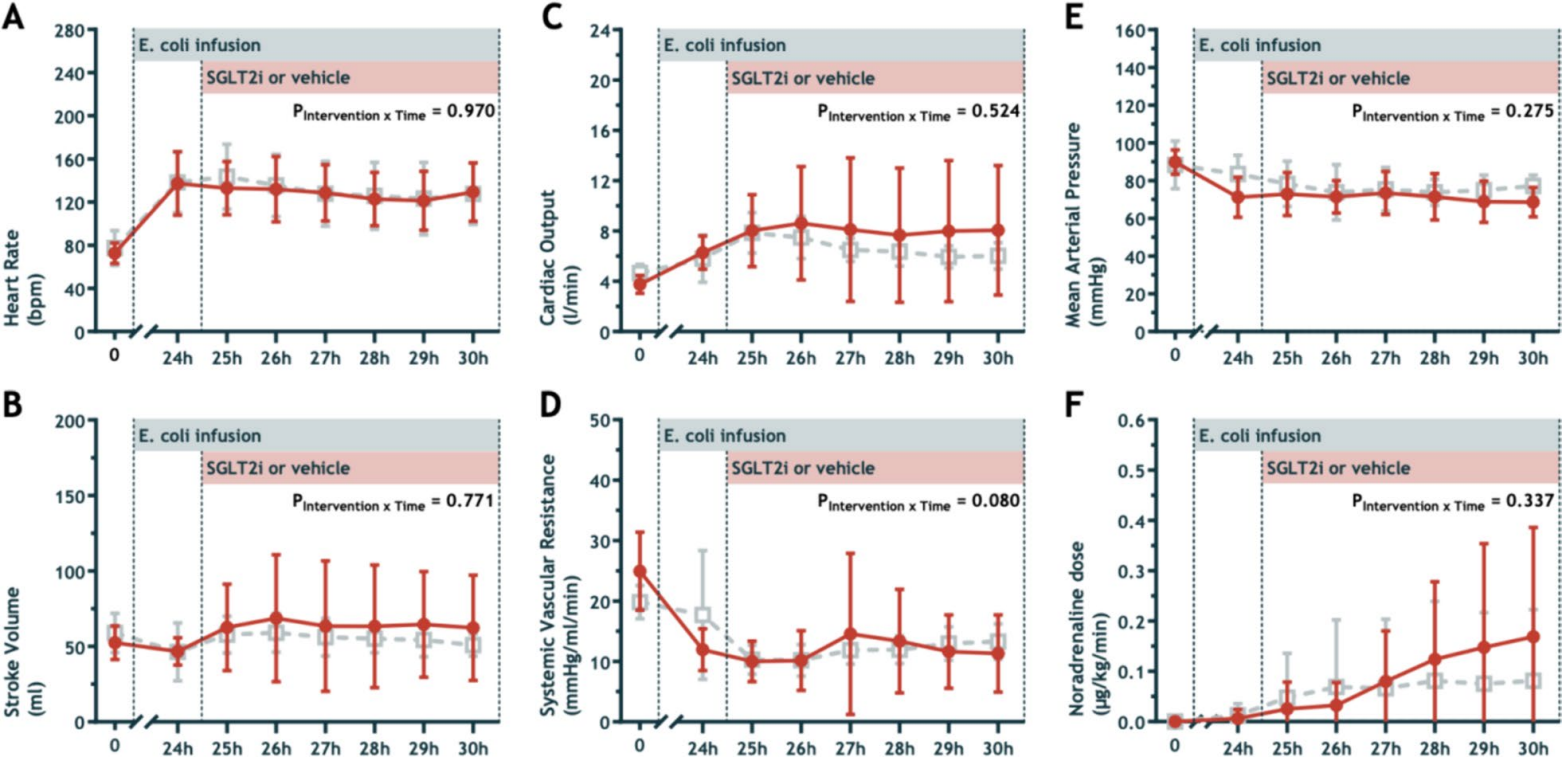
Heart rate (**A**), stroke volume (**B**), cardiac output (**C**), systemic vascular resistance (**D**), mean arterial pressure (**E**) and dose of noradrenaline required (**F**) at baseline and during infusion of E. coli from 0 to 30 h. Sheep treated with an intravenous bolus of 0.2 mg/kg empagliflozin (n = 8) are presented as closed circles and sheep randomized to vehicle solution (n = 8) are shown as open squares. Values are mean ± sd. P-values are outcomes of repeated measures ANOVA

After induction of sepsis and following 24 h of *E. coli* infusion, the sheep developed reduced blood pressure with a hyperdynamic circulatory state (Table 1, Fig. 2). During sepsis induction, heart rate increased and remained stable during the intervention period (Fig. 2A). Stroke volume decreased during sepsis. However, following fluid resuscitation, stroke volume returned to approximately baseline values (Fig. 2B). Systemic vascular resistance decreased after induction of sepsis, and the sheep remained vasodilated during the intervention period (Fig. 2D). The increased heart rate, stable SV, and reduced SVR were accompanied by an increase in cardiac output (3.6 ± 2.0 to 5.3 ± 2.2 L/min) with a decrease in MAP (74 ± 33 to 63 ± 29 mmHg) from baseline to the end of sepsis period. As in the study protocol, noradrenaline was titrated to treat sheep with a target MAP > 65 mmHg (Fig. 2F). During sepsis induction, arterial lactate increased from 0.50 ± 0.13 to 1.36 ± 0.84 mmol/L. The administration of empagliflozin did not result in significant between-group differences in systemic hemodynamics, and the interaction of intervention*time did not reach statistical significance for any of these systemic hemodynamic parameters (Fig. 2A–F).

### Kidney function

**Fig. 3:**
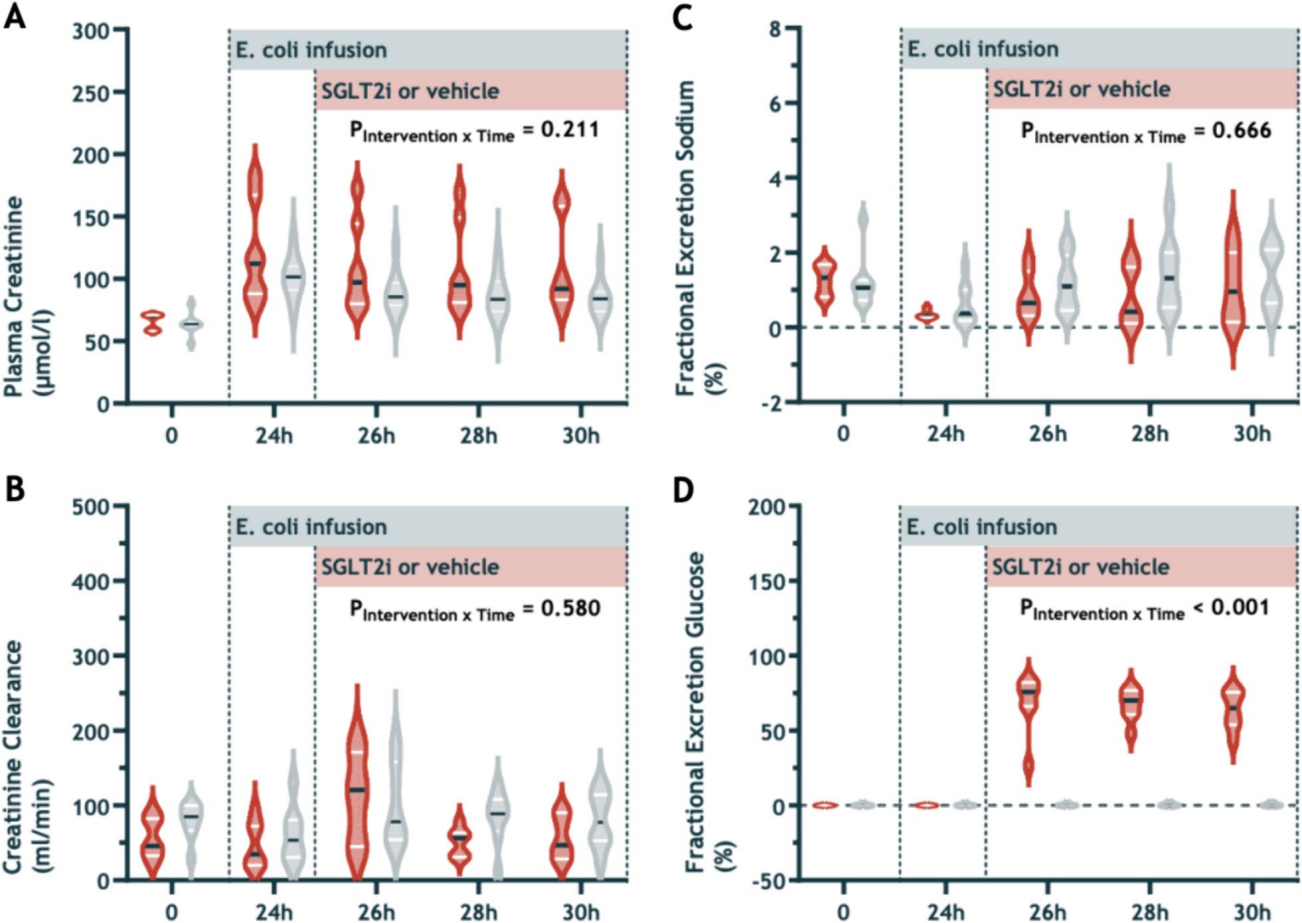
Plasma creatinine (**A**), creatinine clearance (**B**), fractional excretion of sodium (**C**), and fractional excretion of glucose (**D**) at baseline and during infusion of *E. coli* from 0 to 30 h and treatment with intravenous 0.2 mg/kg empagliflozin (red violins, left) or vehicle solution (grey violins, right). Values are mean ± sd

At 24 h after the commencement of *E. coli* infusion, 7 out of 8 sheep in each group had developed stage 1 AKI. Plasma creatinine increased from baseline to the end of sepsis (65 ± 7 to 110 ± 29 μmol/l, p < 0.01) (Table 1, Fig. 3A). Creatinine clearance decreased but did not worsen further during the intervention period (Fig. 3B). During the intervention period, plasma creatinine, creatinine clearance, and fractional excretion of sodium were not statistically different between treatment groups. Fractional excretion of glucose, however, increased significantly in sheep treated with empagliflozin and was substantially greater than the level in the vehicle group (66 ± 2.0% vs 0.14 ± 0.12%, p < 0.001) (Fig. 3D).

### Metabolism

Blood glucose levels remained stable and within normal levels during the study period (at time points: 0 h = 3.0 ± 0.4, 24 h = 2.7 ± 0.9, 30 h = 3.2 ± 1.4 mmol/l). Normal ovine glycemia ranges from 1.4 to 3.6 mmol/l [32, 33]. Glucose (and ketone) measurements are summarized per group in supplementary figure S1, without any between-group differences. Neither sepsis nor the SGLT2i-induced loss of glucose in urine affected glycemia. Ketone measurements revealed no significant ketonaemia or ketoacidosis at any time point in either group. The highest ketone measurement was 0.9 mmol/l (24 h), and the mean at baseline (0 h) was 0.3 ± 0.13 mmol/l.

### Global kidney perfusion and oxygen handling

**Fig. 4:**
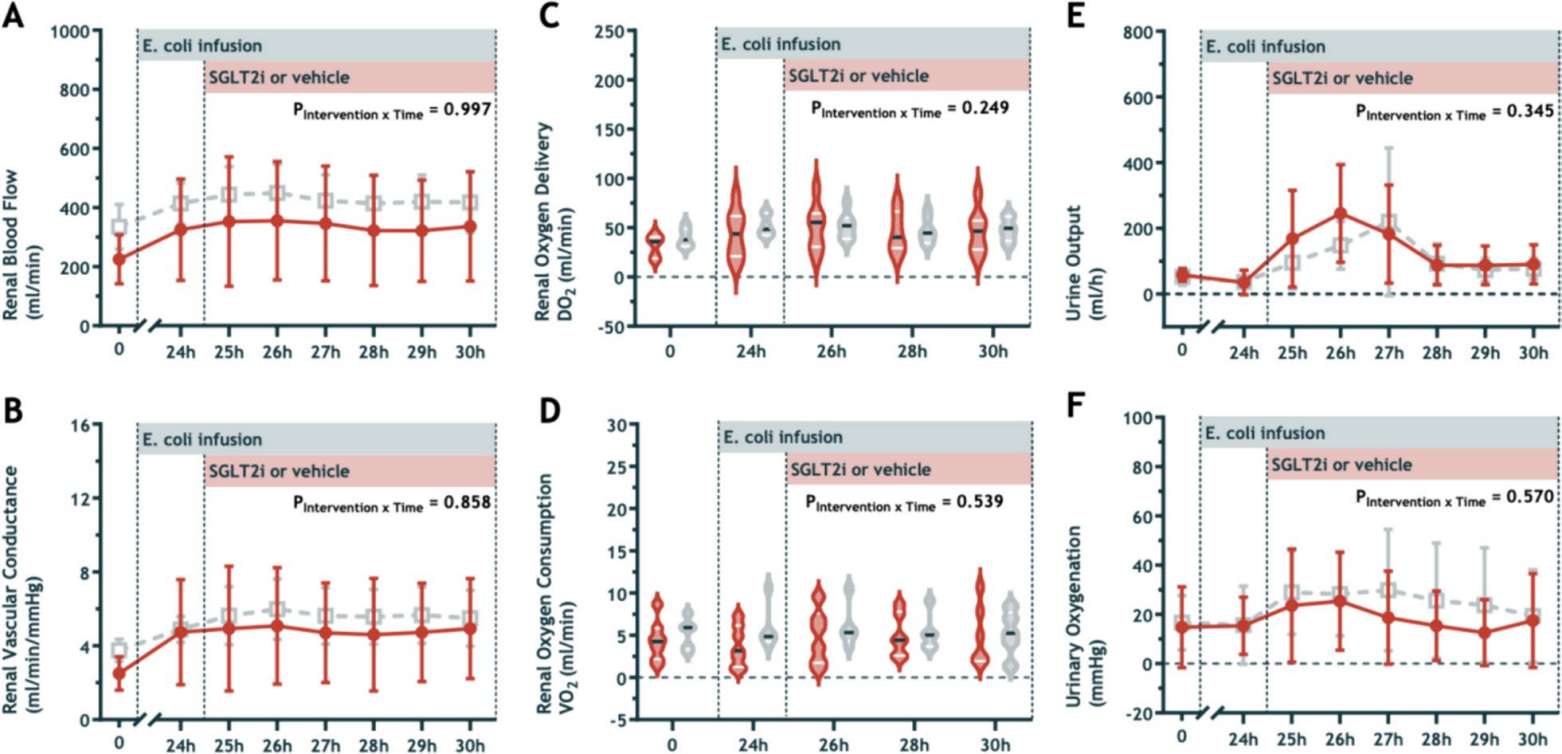
Renal blood flow (**A**), renal vascular conductance (**B**), renal oxygen delivery (**C**), renal oxygen consumption (**D**), urine output (**E**), and, urine oxygenation (**F**), at baseline, during infusion of *E. coli* from 0 to 30h, and during treatment with intravenous 0.2 mg/kg empagliflozin (closed red circles or red violins) or vehicle solution (open grey squares or grey violins). Values are means ± sd

During induction of sepsis, renal blood flow and renal vascular conductance increased, while no between-group differences developed during the intervention period (Fig. 4A and B). We observed no significant alterations in renal oxygen delivery or consumption during the development of sepsis (Fig. 4C and D). By 24 h after live *E. coli* infusion commenced, the urine output rate reduced significantly from the pre-morbid baseline levels (1.39 ± 0.57 to 0.49 ± 0.29 ml/kg/h, P < 0.001). At this time, urine output had dropped to < 0.5 ml/kg/h for > 6 h, consistent with clinical KDIGO criteria for AKI. Urine output (Fig. 4E) increased during the intervention period, following the fluid bolus resuscitation, from 0.9 ± 1.1 to 6.4 ± 4.3 mL/kg/h for the empagliflozin group vs. 0.9 ± 0.3 to 5.4 ± 4.4 mL/kg/h in the vehicle group, (P_Intervention*Time_ = 0.345). We observed a correlated trend in the development of urinary oxygenation (i.e., a temporary increase following fluid resuscitation, Fig. 4F) that was not statistically different between groups.

### Intrarenal perfusion and oxygenation

**Fig. 5:**
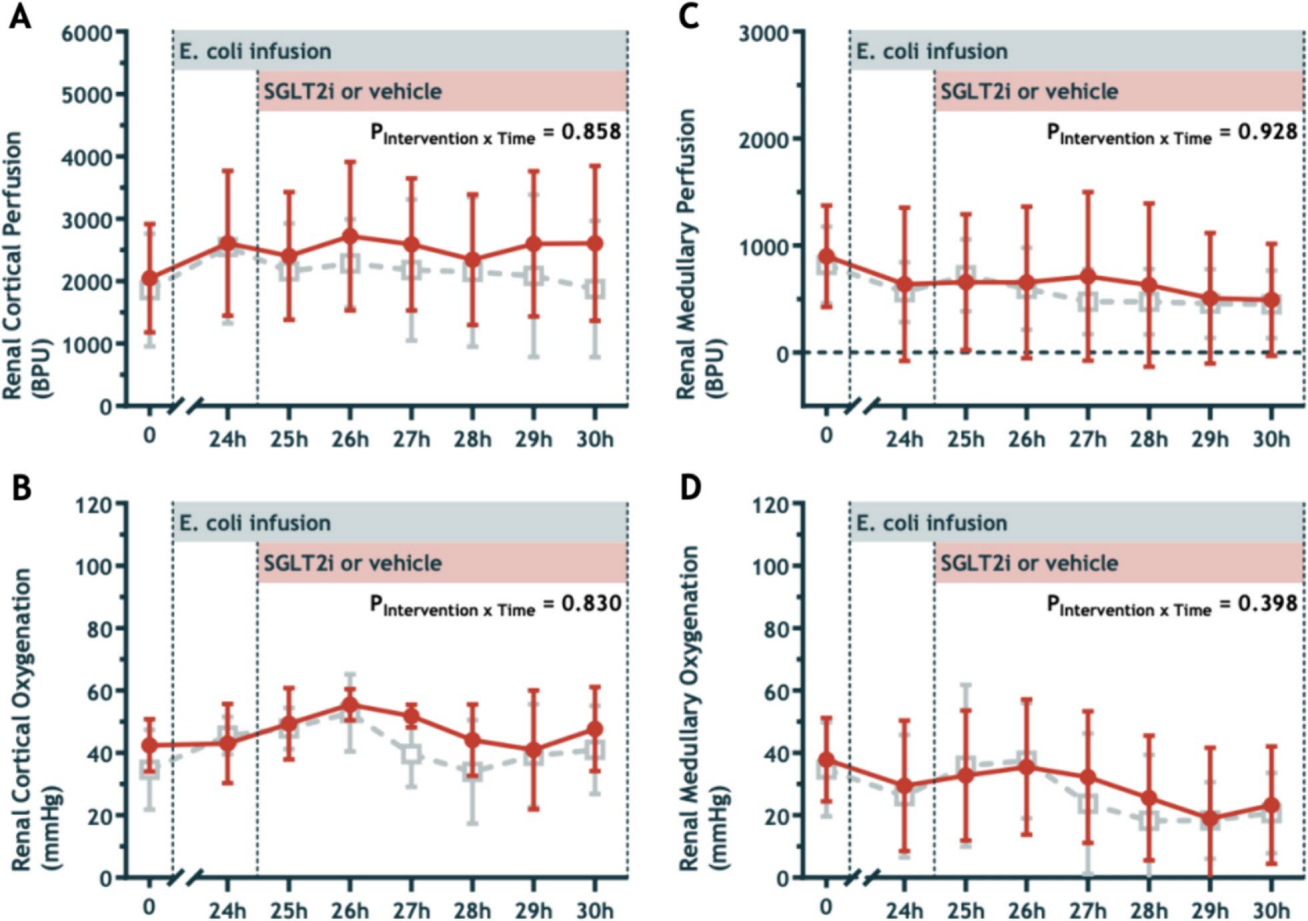
Renal Cortical Perfusion (**A**) and Oxygenation (**B**), and Renal Medullary Perfusion (**C**) and Oxygenation (**D**) at baseline, during infusion of *E. coli* from 0 to 30 h, and during treatment with intravenous 0.2 mg/kg empagliflozin (closed circles) or vehicle solution (open squares). Values are means ± sd

Renal cortical perfusion and oxygenation increased during sepsis, while medullary perfusion and oxygenation decreased (Fig. 5A–D). Renal cortical oxygenation increased following fluid resuscitation (Fig. 5B). We observed no between-group differences in cortical and medullary perfusion or oxygenation during the intervention period. (Fig. 5A–D). Renal cortical oxygenation during the intervention period was 47.6 ± 5.9 mmHg in the empagliflozin group vs 40.6 ± 8.2 mmHg in the placebo group (*P* = 0.16). Renal medullary oxygenation was 28.0 ± 18.5 mmHg in the empagliflozin vs 25.7 ± 16.3 mmHg (*P* = 0.82).

### Renal histopathology

Histopathological examination of renal biopsies revealed diffused tubular injury in two sheep, one from each treatment group (Table 2). Focal changes of tubular injury were present in two sheep in the empagliflozin group and one in the vehicle group. The incidence of tubular injury revealed no significant between-group differences (3/8 vs. 2/8 respectively, *P* = 0.11). Inflammatory changes were present in three of the sheep in each treatment group. Tubular casts were seen in 3/8 in the SGLT2i vs. 5/8 in the vehicle group (*P* = 0.32). Between-group comparisons of inflammatory changes and the presence of tubular casts revealed no significant difference between the SLGT2i and placebo groups (Table S1).

**Table 2:** Renal pathological changes in empagliflozin and vehicle-treated groups

	Empagliflozin (n = 8)								Vehicle (n = 8)							
	E1	E2	E3	E4	E5	E6	E7	E8	V1	V2	V3	V4	V5	V6	V7	V8
Tubular injury	+	0	+	0	0	0	0	++	0	0	+	0	0	0	++	0
Interstitial inflammation	+	0	+	0	0	0	0	++	++	0	+	0	0	0	++	0
Tubular casts	+	+	0	0	0	0	0	++	0	0	+	++	0	++	+	+

Zero (0), no histological renal tubular injury, inflammation, or tubular casts; ( +) = mild or focal histological renal tubular injury, inflammation, or tubular casts; (++) = significant histological renal tubular injury, inflammation, or tubular casts

## Discussion

In an ovine model of Gram-negative SA-AKI, we studied the acute effects of SGLT2 inhibition on systemic hemodynamics, renal function, and intrarenal perfusion and oxygenation. We showed that inhibition of SGLT2 did not induce renoprotection in established ovine SA-AKI, as indicated by the lack of improvement in systemic hemodynamics, renal and intra-renal perfusion and oxygenation, and kidney function. Histopathological examination of kidneys at necropsy found no adverse effects of SGLT2 inhibition in ovine SA-AKI.

### Relationship to previous studies

The hypothesis that SGLT2i would be beneficial in SA-AKI was based on the findings from large cardiovascular outcome trials in patients with type 2 diabetes, heart failure, and chronic kidney disease [12, 14, 34]. Meta-analyses of these trials showed that SGLT2i treatment reduced the relative risk of AKI by 66–81% [12, 14, 34]. An important difference with our study is that these patients received long-term and repeated treatment with an SGLT2i, and the incidence of AKI was lower during a multi-year follow-up period. It is plausible that a single dose of SGLT2i in established SA-AKI was not similarly effective despite an immediate increase in glucosuria following empagliflozin administration.

A second important difference is the time of treatment initiation, which distinguishes between preventive and treatment interventions. Chronic SLGT2i use ensures effective plasma SGLT2i concentrations are achieved before an index event with the potential to cause AKI (such as illness or surgery). In this study, SGLT2i was administered as a treatment at 24 h of established sepsis when 14/16 sheep had already met the KDIGO criteria for Stage 1 AKI. The findings of the randomized placebo-controlled DARE-19 study support the suggestion that treatment after the development of illness is potentially less effective. The DARE-19 study investigated whether treatment with dapagliflozin could protect patients with cardiometabolic risk factors from additional organ injury when hospitalized with COVID-19 [35]. In this study, dapagliflozin did not prevent the primary endpoint of new or worsened organ dysfunction or death, nor any of the secondary endpoints, including kidney failure. Of note, this study excluded critically ill patients requiring ICU admission.

Our study focused on the possible kidney protective effects of SGTL2i mediated through changes in renal and intrarenal perfusion and oxygen handling. As SGLT2 inhibition reduces the metabolically demanding process of glucose reabsorption, it is hypothesized that it would also reduce renal oxygen consumption and increase tissue oxygenation. In support of this hypothesis, SGTL2i reduced kidney oxygen consumption and reversed renal cortical hypoxia in control and diabetic rats [36]. This finding was confirmed in a clinical trial of adults with diabetes mellitus and albuminuria. Blood oxygenation level-dependent (BOLD) MRI revealed that a single dose of dapagliflozin increased renal cortical oxygenation without affecting renal blood flow or renal tissue perfusion [17]. In our ovine model of SA-AKI, we did not observe a between-group difference in cortical oxygenation during the intervention period. While consistent with previous studies from our group, ovine septic AKI was characterized by renal medullary tissue hypoxia with preservation of renal cortical oxygenation [37]. As such, it is conceivable that SGLT2 inhibition may be more effective in acute or chronic kidney diseases that are characterized by renal cortical tissue hypoxia. Importantly, our study found no adverse effects of SGLT2i on renal histopathology in established ovine SA-AKI.

### Strengths and limitations

Our clinically relevant large animal model of SA-AKI has been studied extensively [21, 22, 25, 27, 28]. The model closely resembles the human hemodynamic and renal physiology during the early stage of sepsis, at least over 24 to 48 h of live infection. The study methodology was designed with clinical practice in mind. Sepsis was induced over 24 h without other interventions; then, at a time analogous to presentation to a hospital, the sheep received fluid resuscitation, after which the intervention was administered. Group allocation was based on randomization, and histopathological analyses were performed by an experienced pathologist blinded to treatment group allocation. As such, we studied the effects of SGTL2i in a clinically relevant model of established septic shock and developing AKI. However, our treatment included a single dose of empagliflozin, and our hemodynamic monitoring period was limited to 6 h (from 24–30 h of sepsis). While SGLT2i has beneficial cardiovascular and renal effects in patients with diabetes and heart failure, we cannot comment on whether that lack of effect in SA-AKI was due to the short treatment duration. Assessing the state and severity of sepsis in an animal model can be difficult, especially compared to clinical experience. However, the degree of organ dysfunction reflected by increases in cardiac output and lactate levels or decreases in arterial oxygenation and urine output are comparable to previous studies and reflect a state of early sepsis and developing organ dysfunction. Comparison to clinical practice is likewise limited by the experimental induction of sepsis through the intravenous administration of *E. coli* in contrast to a primary organ focus of sepsis*.* In contrast with clinical practice, we studied young female sheep without known comorbidities.

## Conclusion

In a large animal model of gram-negative sepsis-associated AKI, we could not demonstrate kidney protective properties of SGLT2i on systemic hemodynamics, (intra-)renal perfusion, and oxygenation. We cannot exclude that an earlier intervention or more prolonged treatment with an SGLT2i could reduce AKI. However, the context of sepsis demands treatment after the fact, precluding a preventive approach. To explain our negative findings, we consider that previous evidence of the renoprotective effects of SGLT2i was related to improvements in renal cortical oxygenation, whereas our ovine model of sepsis-associated AKI is typically characterized by renal medullary hypoxia, making this a less promising target for SGLT2i.

### Supplementary Information

Supplementary Material 1.

## Data Availability

Data are available upon request to the corresponding author.
