# Supplementary material for: Effects of sodium-glucose transporter-2 inhibition on systemic hemodynamics, renal function, and intra-renal oxygenation in sepsis-associated acute kidney injury
Source: Intensive Care Med Exp. 2024 Jul 8;12:64. doi: 10.1186/s40635-024-00647-2 (PMC11231125; doi:10.1186/s40635-024-00647-2)
Supplement: Supplementary file 1 — Supplementary Material 1. [file 40635_2024_647_MOESM1_ESM.docx]

**Table S1.** Number of sheep (out of 8) that satisfied the criteria for each of the histopathological abnormalities observed in each treatment group.

| Scoring criteria | **Acute tubular necrosis** | **Inflammatory cells present** | **Interstitial fibrosis** | **Tubular casts** | **Red blood cells** |
| --- | --- | --- | --- | --- | --- |
| **Empagliflozin** | | | | | |
| - negative | 5 | 4 | 8 | 5 | 5 |
| +/- focal | 2 | 3 | 0 | 2 | 0 |
| ++ diffuse | 1 | 1 | 0 | 1 | 3 |
| **Vehicle-** **ß-cyclodextrin** | | | | | |
| - negative | 6 | 4 | 8 | 5 | 6 |
| +/- focal | 1 | 3 | 0 | 2 | 1 |
| ++ diffuse | 1 | 1 | 0 | 1 | 1 |

Sections of the renal cortex and medulla were collected at autopsy following 48 h of recovery in sheep that received either empagliflozin (N=8) or vehicle treatment (N=8) during gram-negative sepsis. Formalin-fixed paraffin-embedded renal tissue was subjected to histochemical and immunohistochemical analysis for acute tubular necrosis (focal or diffuse denudation and/or flattening of tubular epithelial cells with tubular denudation, interstitial edema, loss of proximal tubular brush borders and tubular casts), interstitial mononuclear infiltrates (peritubular), fibrosis (interstitial), casts (hyaline, cellular or granular) and birefringence (oxalate) crystals. The grading system used includes Grade 1 (0) = no change compared with morphology on kidneys collected from healthy sheep; Grade 2 (+/-) = Focal change in 5-30% of total section; Grade 3 (++) = Focal change in 30-50% of the total section.

**Figure S1.**


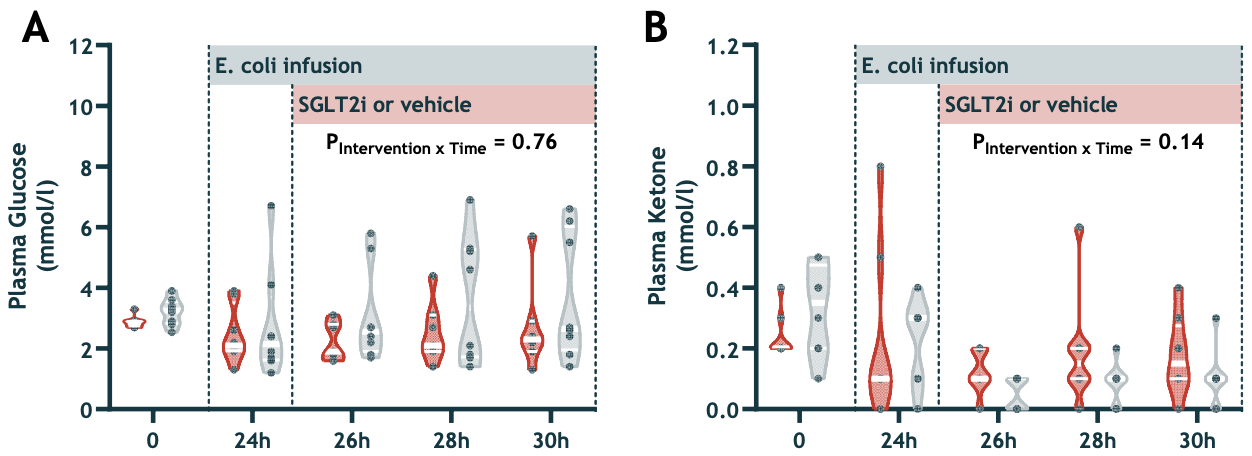


**Figure S1.** Plasma Glucose (A) and Ketone (B) measurements at baseline, during infusion of E. coli from 0 to 30h, and during treatment with intravenous 0.2 mg/kg empagliflozin (closed circles) or vehicle solution (open squares). Values are means ± sd.
